# Carate, a Non Classified Disease of the Skin

**Published:** 1859-04

**Authors:** 


*Carate, a non-classified Disease of the Skin.* By Dr. G. Van Arcken.

The name of this disease, of which the author has failed to find any description in dermatological works, is derived from *cara*, the Spanish for *face*, and *ate*, the Indian for *look*—the word, according to Dr. Van Arcken, meaning "Look at his face." It occurs in New Granada and the northern parts of South America, and presents three varieties—the blue, the white, and the rose-colored. The *blue* is the mildest, attacking persons of fifteen to twenty-five years of age, and consisting in the appearance of blue, round or oval spots on the face. The spots coalesce, and extend down the neck on to the chest, where the ribs are often so distinctly marked as to cause the patient to resemble a zebra. The hands are a favorite seat of the disease, and again, the lower end of the tibia. It sometimes appears on the glans penis; but the female organs of generation remain free. The *white* variety rarely occurs in the male; it is commonly accompanied by diseases of the ovaries and uterus. The color is of a dead chalky white, and attacks people of from thirty to forty years. The *rose-colored* variety is the worst kind, and frequently follows the white, in which case there appears on the chalky spots of the latter some very minute red spots, which gradually enlarge, until the whole assumes a pale red color. Those affected with this disease are mostly Sambos, Mulattoes, and others of a dark complexion. It always commences on the hands, extends from them to the face and neck, and then down over the abdomen. It attacks both sexes equally. Dr. Van Arcken attributes the disease to a combination of such influences as filth, exposure, syphilis, and unwholesome food. Syphilis prevails so extensively, that scarcely one in a thousand inhabitants is free from it. It seems, therefore, to be a necessary complication of every disease occurring in those parts. Carate is regarded as incurable by the native practitioners; Dr. Van Arcken has found alterative mercurial treatment, iodides, and arsenic, successful remedies. The blue variety he cures in about eight weeks; the other two forms require a longer period; "but the complete cases, whether they be congenital or contracted afterward, are better left alone." Carate is never fatal by itself.—*Ranking's Half-Yearly Abstract of Medical Sciences.*

